# Ensemble automated approaches for producing high‐quality herbarium digital records

**DOI:** 10.1002/aps3.11623

**Published:** 2024-11-05

**Authors:** Robert P. Guralnick, Raphael LaFrance, Julie M. Allen, Michael W. Denslow

**Affiliations:** ^1^ Florida Museum of Natural History University of Florida Gainesville Florida USA; ^2^ Department of Biological Sciences VirginiaTech Blacksburg Virginia USA

**Keywords:** ChatGPT, digitization, ensemble methods, information extraction, large language models, machine learning, natural history collections, natural language processing

## Abstract

**Premise:**

One of the slowest steps in digitizing natural history collections is converting labels associated with specimens into a digital data record usable for collections management and research. Here, we address how herbarium specimen labels can be converted into digital data records via extraction into standardized Darwin Core fields.

**Methods:**

We first showcase the development of a rule‐based approach and compare outcomes with a large language model–based approach, in particular ChatGPT4. We next quantified omission and commission error rates across target fields for a set of labels transcribed using optical character recognition (OCR) for both approaches. For example, we find that ChatGPT4 often creates field names that are not Darwin Core compliant while rule‐based approaches often have high commission error rates.

**Results:**

Our results suggest that these approaches each have different strengths and limitations. We therefore developed an ensemble approach that leverages the strengths of each individual method and documented that ensembling strongly reduced overall information extraction errors.

**Discussion:**

This work shows that an ensemble approach has particular value for creating high‐quality digital data records, even for complicated label content. While human validation is still needed to ensure the best possible quality, automated approaches can speed digitization of herbarium specimen labels and are likely to be broadly usable for all natural history collection types.

The natural history collections community has made enormous progress in large‐scale digitization of specimens over the past two decades, catalyzed by a series of technical and social advancements (Hedrick et al., 2020). However, label digitization, a process that converts analog information on labels into digital text, which can then be atomized into proper fields in digital databases, remains one of the slowest steps in overall workflows (Guralnick et al., 2024). This step has remained slow because it has required significant human input to deliver high‐quality results, even when collections employ some automated steps, such as label optical character recognition (OCR).

Automated approaches hold promise to help speed label digitization (Takano et al., 2024). The goal of such approaches is to take an image of a label and return a high‐quality output conforming to a standardized specimen record, e.g., conforming to the Darwin Core standard (Wieczorek et al., 2012; Figure 1). Unfortunately, all steps of this process often still produce relatively high error rates, such that efforts needed to correct mistakes are time‐costly (Guralnick et al., 2024). For there to be broad‐scale uptake of new automation approaches, they must be significantly better and faster than what can be achieved via human effort. Very recently, new machine learning approaches, especially large language models (LLMs), hold promise to dramatically improve some steps of this process, especially atomizing text into standardized fields (Weaver et al., 2023; Figure 1).

**Figure 1:**
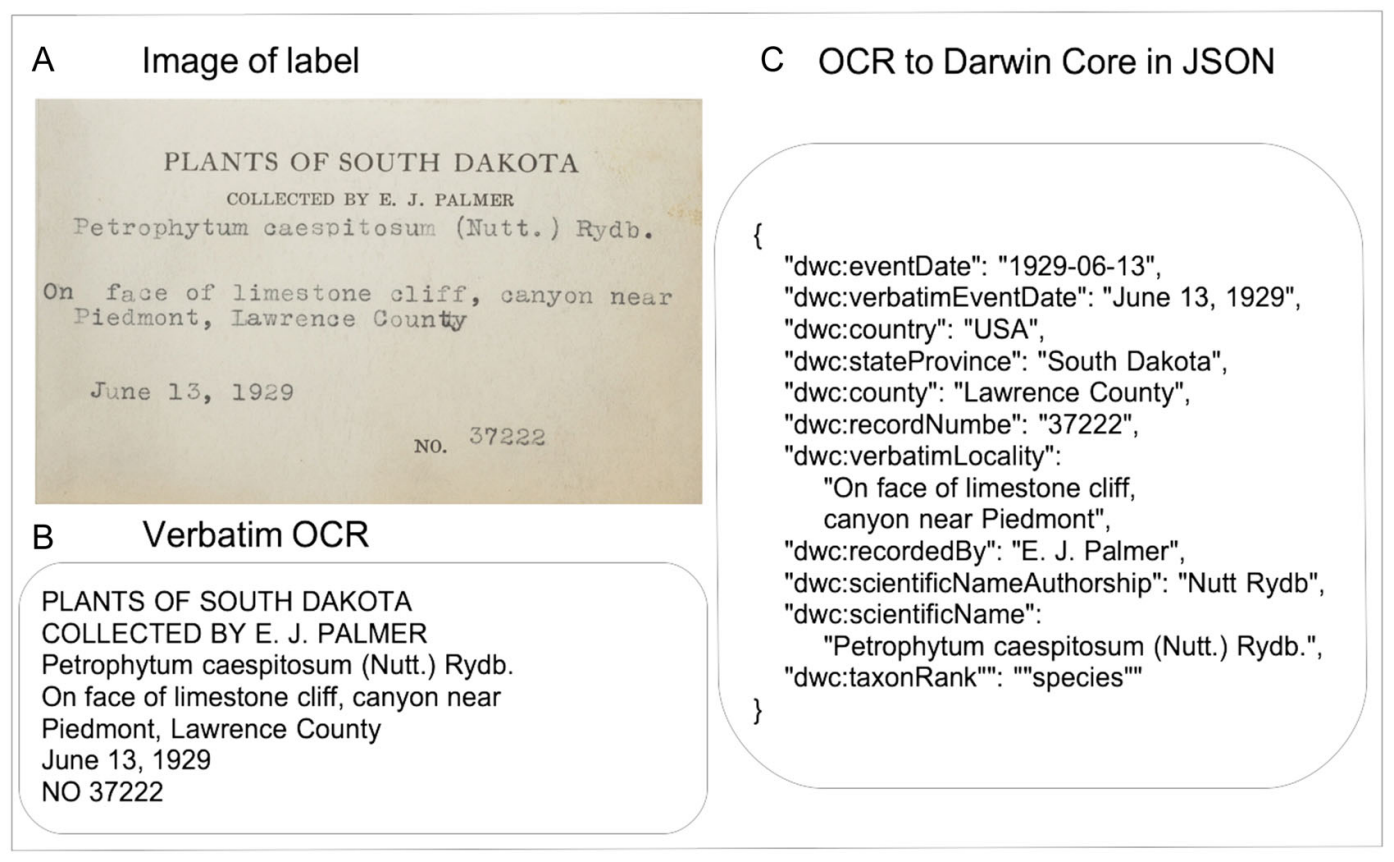
Example showing the goal of automated label digitization via the conversion of (A) label text to (B) digitized text by means of optical character recognition (OCR), which is then converted from verbatim text to (C) JSON‐formatted parsed data in Darwin Core format.

Despite their enormous potential, the degree to which new tools such as LLMs (e.g., ChatGPT4; OpenAI et al., 2023) can enhance the quality and speed of herbarium label digitization is just beginning to be explored (Weaver et al., 2023). Furthermore, there are other approaches that utilize natural language processing (NLP) (see Owen et al., 2020) that have yet to be fully tested and compared to LLM approaches. For example, rule‐based NLP (RB‐NLP) has been used in many different applications in text mining biological and other types of data (Xu and Cai, 2021) and may prove to be a more reliable alternative. The key questions yet to be fully addressed involve the error rates associated with different approaches, and if and how those approaches can be combined to further improve results.

Here we provide a detailed assessment of how well different approaches work for atomizing OCR text from herbarium labels into Darwin Core fields, a standard widely used by the natural history collections community. We do so by first providing details on the development and performance of a RB‐NLP information extraction approach, comparing it directly to results from queries that were engineered to best enable extracting and atomizing label data using ChatGPT4. We calculate omission and commission error rates for both tools, focusing on core target fields that are essential to capture from labels. Finally, we showcase an ensemble approach that combines rule‐based and ChatGPT outputs, which performed far better than either approach separately. Our overall work provides an assessment of what is possible, keeping in mind that we are just at the start of what is likely to be a major transition from human transcription to more efficient automated approaches in label digitization efforts.

## METHODS

### OCR test data

We used a set of label data digitized via OCR from Guralnick et al. (2024) as a test set. All of the labels came from the Global Biodiversity Information Facility (GBIF; https://www.gbif.org/). We searched the GBIF database in September 2023 for all specimens meeting the following criteria: members of Tracheophyta, collected in the United States, record containing a specimen image, preserved specimen type, English language, and not cultivated. This search resulted in 4,091,778 records, of which we randomly selected 2128 typewritten labels, most of which were from the main label on the specimen sheet. Here we define a main label (versus a determination label or other kind of label) as the label that contains the data and metadata that usually compose the key specimen record information such as taxon, collector, locality, and date.

OCR was performed on these labels using a custom pipeline that was described in detail in Guralnick et al. (2024). This pipeline (https://github.com/rafelafrance/ocr_ensemble) has a set of pre‐ and post‐processing steps that improve quality over off‐the‐shelf open‐source solutions such as Tesseract (Kay, 2007; https://github.com/tesseract-ocr/tesseract). The OCR content was not corrected prior to being used in downstream workflows, because the goal was to determine the success of parsing when there are an unknown number of OCR errors in the input. However, as we discuss below, when validating our outputs, we ignored cases that were not main labels (typically these were longer determination labels that were not caught in our pipeline) or those where OCR was so poor that it was impossible to evaluate parsing quality.

### RB‐NLP development

**Figure 2:**
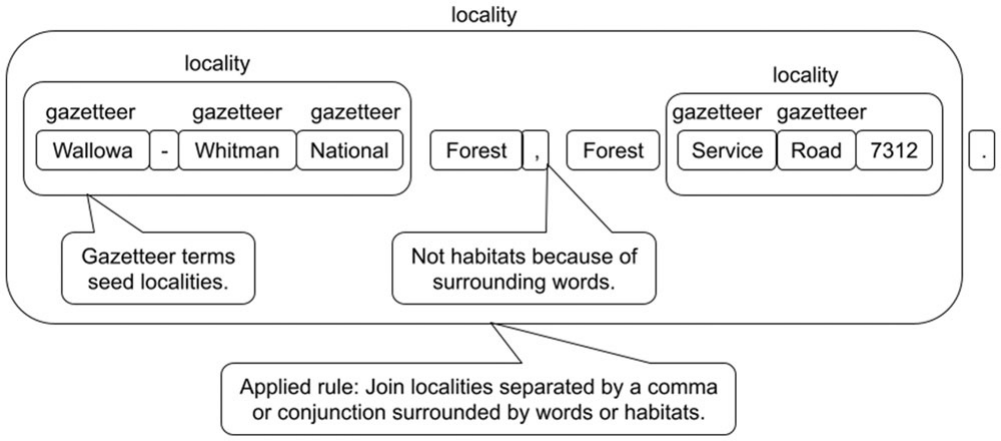
Example of a rule‐based parsing approach to discover locality descriptions within a label. The LRBE starts by using a gazetteer to find key works that seed the locality. A set of rules are used to extract all the content that belongs in the field “dwc:locality”. For example, in this case, “Forest” is not a habitat term in either usage above, because in the first case it is tied to the preceding word “National”, which is a place name, and in the second case it is followed by “Service”, which implies a type of road. Different locality pieces are joined based on an applied rule to create a final output string labeled as “locality”.

Our label RB‐NLP extraction (LRBE) approach uses a multistep approach to extract text and link to named Darwin Core terms. The rules themselves are written using spaCy (Honnibal and Johnson, 2015), which is a key NLP library in the Python programming language, with enhancements we developed to streamline the rule‐building process. These rules focus on existing models in spaCy that have been developed for breaking text into tokens, building phrases and more complex content, and extracting the results. The general outline of the rule‐building process follows:

1. We assembled Darwin Core terms that need to be associated with parts of OCR content. We then used existing term labels already formatted in Darwin Core format from iDigBio (https://www.idigbio.org/) and other sources with a set of content terms associated with those labels.
2. We also assembled some key corpi (e.g., names of known plant taxa from Kew Plants of the World Online [https://powo.science.kew.org/], the World Flora Online plant list [Borsch et al., 2020], and the Integrated Taxonomic Information System [ITIS] database [https://www.itis.gov/]) to help with matching to key fields (e.g., dwc: scientificName).
3. We used this expert content to develop spaCy's phrase matchers. These are rarely sufficient for capturing the content needed, especially for more complex content such as locality or habitat, so we used phrase matches as anchors for extracting more complex content.
4. We then built more complex content from the simpler matched phrases using spaCy's rule‐based matchers repeatedly (Figure 2). We linked related content together via defined entity relationships using spaCy rules.

In developing the LRBE approach, we iteratively tested the performance of the tool on the same set of OCR labels. This involved two of the authors (R.P.G. and M.W.D.) examining hundreds of already formatted labels, identifying common problems with extraction quality, and then determining whether there was a rule that could be added to improve the results. After multiple iterations, we were able to remove many potential issues and improve parsing, but the challenge remained that the LRBE often produced extracted content with both commission and omission errors. A commission error is a case where there is extra content in a field that is clearly not correct and belongs in another field. Omission errors are cases where the extraction approaches missed content that was clearly supposed to be in the field in question. We quantified those errors and compared them with rates from ChatGPT4 and an ensemble approach, as described below.

### Using ChatGPT for label parsing

In contrast to LRBE, LLMs like ChatGPT require very little knowledge of how they work and most of the upfront effort involves “prompt engineering.” Prompt engineering is shaping your queries to the LLM so that they yield the best results possible, which is an art form in itself. Our approach to prompt engineering was to keep the prompts small and focused on extracting information in Darwin Core format. The prompt that we used for this paper was: “You are an expert botanist. Extract all information from the herbarium label text and put the output into JSON, a compact text format, using Darwin Core fields including dynamicProperties.” This was followed by the same OCR label text used for testing the LRBE approach. This is a small prompt that worked reasonably well. We used the application programming interface (API) for gpt‐4‐0613 with standard settings for temperature, “top_p”, frequency penalty, presence penalty, and maximum tokens to retrieve results.

### Developing an ensemble approach

ChatGPT4 and the LRBE approach each had their own strengths and weaknesses. Because of this, we opted to ensemble the two approaches with the goal of utilizing the best of both approaches to reduce error rates. Doing so involved a process of reconciling outputs across the same or similar fields to a consensus output. We refer to the overall process as “ensembling,” but within a field or set of fields, we call this process “reconciliation.” With some exceptions, there is one reconciler per Darwin Core term. Most reconcilers are very simple, prioritizing either ChatGPT or LRBE outputs and making sure that those align with known Darwin Core terms. Others are a bit more complex, as we discuss below. Each reconciler takes as input the JSON data from LRBE and from ChatGPT that was subsequently cleaned, and the original label text that was fed to both.

One of the most challenging problems with ChatGPT4 outputs is that it attempts to extract data into Darwin Core terms but will often create field names for content it cannot fit in Darwin Core, some of which could be mapped to known Darwin Core terms. This led to a surprising profusion of terms that are not in the Darwin Core–controlled vocabulary, discussed more below in the Results section. To handle these issues, we created aliases for all the reconcilers including non–Darwin Core terms that map them to the correct term. For example, ChatGPT created the terms “dwc:locationState”, “dwc:state”, and “dwc:province”, which we aliased to the proper term “dwc:stateProvince”.

An example of a challenging Darwin Core field to reconcile is “dwc:verbatimLocality”. Before developing the reconciler for this field, we noted a few observations: (1) ChatGPT tends to correctly find the locality more often than LRBE, but when LRBE finds the correct locality it often finds a longer correct version. This longer version is often broken up into a list of locality phrases rather than one contiguous locality value. (2) ChatGPT sometimes puts a separate locality notation under the “dwc:locationRemarks” term. (3) ChatGPT's version of locality is sometimes presented as a nested object. That is, it is itself a dictionary of locality‐related terms that need to be assessed and reconciled.

Given the above, the process we use to reconcile “dwc:verbatimLocality” is as follows. First, we look for the locality in the ChatGPT output listed under any of its aliases and in dwc:locationRemarks. If ChatGPT's version of locality is a nested object, then we try to pull an accurate locality from one of its sub‐terms. If that is not possible, we then use the LRBE's version. When examining the LRBE's version, we focus on Darwin Core locality fields because LRBE does not have the same issues as ChatGPT with inventing Darwin Core fields. If the LRBE content is a contiguous list of partial localities, we composite that list into a seamless single string. In cases where ChatGPT locality (or aliases) are present, we still check in the LRBE version, and if the ChatGPT locality is completely contained within a larger LRBE locality string, we then use the LRBE version. Finally, we check to see if content in the ChatGPT location remarks can be used to either extend the currently used locality or use it as another item in a locality list.

### Testing error rates for LRBE, ChatGPT4, and ensemble approaches

We randomly selected 200 total outputs from LRBE and ChatGPT, skipping labels that were not the main labels or where OCR was so poor that the labels were effectively illegible. The authors (M.W.D. and R.P.G.) then scored error rates for a total of 100 of the 200 as follows. We defined a set of core fields that are often of particular importance to properly capture and are present on a majority of labels. These fields are: recordedBy, recordNumber, eventDate, locality, country, stateProvince, county, and scientificName. We explicitly captured information on the number of commission and omission errors in target fields in order to determine the performance of the text extraction tools.

We utilized a rubric for scoring errors across LRBE, ChatGPT, and our ensemble approach as follows. First, we explicitly skipped determination labels as well as labels where OCR results were so poor as to significantly impact the ability of either LRBE or ChatGPT4 to work effectively. We did not, however, clean OCR outputs except to remove the worst OCR outputs that were effectively illegible, because the goal here is to see how well these approaches perform in automated pipelines where there is likely to be a low percentage of OCR issues remaining during the parsing step. Second, if data for a target field were missing from OCR of the label, we scored the target as “NA”. Third, we were not concerned with semantic interpretations, such as expanding a country name from “USA” to “United States of America”, or date reformatting. We also did not flag commission errors for cases where the locality contained county or state information, as long as it was also captured in the correct fields, nor did we note an error when ChatGPT added an instance of higher geography (e.g., country) when it was not on the label, unless it was clearly a mistake. We then tabulated error rates for each target field individually and also calculated overall error rates for each information extraction approach. Finally, we addressed whether the length of the label explains the potential for errors in the ensemble approach, which we would expect because more content should mean more chances for either approach and ultimately the ensemble to mis‐assign content, thus leading to errors in reconciled outputs. We simply fit a single predictor model with the length of the label, measured as total number of characters, as a predictor of whether the record had an error (or errors), using the *glm()* function to fit a logistic regression in base R (R Core Team, 2021).

### Testing ChatGPT4 Darwin Core field names

ChatGPT does not always return canonical Darwin Core field names published as part of the standard (https://dwc.tdwg.org/terms/). In order to quantify the magnitude of this problem, we counted how many times ChatGPT used a non‐canonical field name from our full record set. We counted, in particular, how many unique field names that ChatGPT “invented” or “hallucinated” that were non‐canonical and the number of records and fields that were impacted across all the labels. Finally, for the core fields defined above, we determined how many synonyms existed that linked to the proper core field name.

## RESULTS

### Summary of performance within core fields

The commission and omission error rates for the LRBE, ChatGPT4, and ensemble approaches for the core fields (defined above in the Methods) are summarized in Table 1. The key finding is that ChatGPT performs much better than rule‐based parsing in terms of omission and commission errors, with nearly two‐fold fewer errors than a rule‐based approach. ChatGPT4 is particularly good in not making commission errors, with a nearly 10‐fold improvement in commission compared to LRBE. In the LRBE approach, multiple dates and especially numbers (such as road numbers in locality descriptions) often ended up wrongly linked to eventDate or recordNumber, leading to a much higher rate of commission. Totals by target fields for each approach are shown in Tables S1 and S2 (see Supporting Information).

**Table 1:** Error counts for ChatGPT4, label rule‐based NLP extraction (LRBE), and ensembled results based on scoring of 100 randomly selected herbarium records. These are errors for essential Darwin Core fields when present (recordedBy, recordNumber, eventDate, locality, scientificName, country, stateProvince, county).

Method	ChatGPT4	LRBE	Ensemble‐based
Commission errors	7	93	22
Omission errors	92	100	46
Total	99	193	68

**Figure 3:**
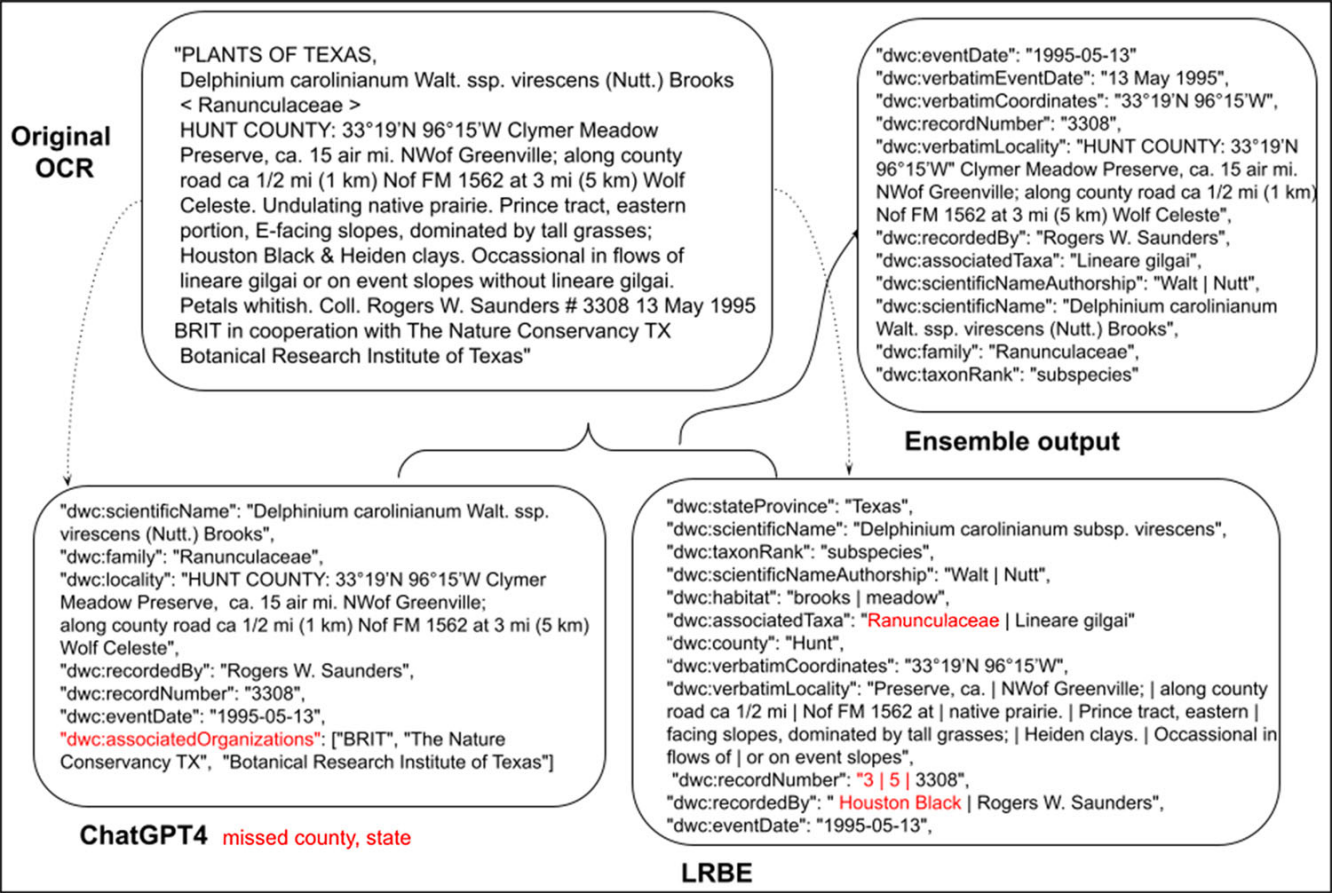
Examples of the workflow and processing outputs from an exemplar label, starting with the original OCR text (top left), the ChatGPT4 output (bottom left), the LRBE output (bottom right), and the ensemble output (top right). We have excised any dwc:dynamicProperties content returned by either extraction method. Errors in extraction are shown in red, with omissions shown below the label contents. In the ChatGPT4 output, dwc:associatedOrganizations is an example of a hallucination as this is not a Darwin Core term.

Ensemble methods performed the best of all, significantly reducing omission errors compared to ChatGPT4. The decrease in omission rates and improvement in reducing non–Darwin Core terms is due to complementarity and leveraging what each tool does best. LRBE is less likely to hallucinate terms while ChatGPT is better at extraction, but one or the other will often do better on different labels. The ensemble approach often captures the best outcomes, although it has a slightly higher rate of commission than ChatGPT. The ensemble approach also performed markedly better on another key metric—the number of records (out of 100) with no errors; the ensemble approach had 50% of records with no errors, ChatGPT had 37% with no errors, and LRBE had 15% without errors. An example output from both ChatGPT4, LRBE, and the ensemble approach (Figure 3) nicely illustrates issues with ChatGPT4, including omission errors and mistakes in Darwin Core field names, and commission issues with LRBE; all of these problems are resolved in the ensemble output. Further discussion of the performance of the ChatGPT4, LRBE, and ensemble approaches is below.

To determine if the length of a label affects error rates for our ensemble method, we ran a logistic regression, which showed that label length is a (marginally) significant predictor of at least one error (*P* < 0.035) and longer labels do have higher odds of at least one error (label length coefficient estimate: 0.002055) but with moderate uncertainty (standard error: 0.000972). On average, the odds of an error increase from about 40% for a short label (~250 characters) to about 68% for longer ones (>750 characters) (see Figure S1).

Finally, we also examined ensemble error rates across core fields (Table 2). We expected that locality would have the highest error rates by a large margin, given how difficult this content can be to extract as it is more free‐form and complex than other content on labels. We found that, while locality does have more errors than other fields, the differential is much smaller than expected, and the ensemble‐based method is surprisingly good at assembling coherent locality information from these records. Some fields, such as locality and recordedBy, mostly had omission issues, likely due to relying significantly on ChatGPT (which is high in omission and low in commission), while others, such as dwc:stateProvince, primarily had commission errors. In fields where there are high commission rates in the ensemble outputs, missing content in ChatGPT was often filled in with LRBE content, which often led to increased commission (see Tables S1 and S2).

**Table 2:** Types of core field errors contained in reconciled output. We note 68 errors in total. Not all core fields are present in each record. We use Darwin Core names for field names.

	Darwin Core field							
Error type	recordedBy	recordNumber	eventDate	locality	scientificName	country	stateProvince	county
Commission errors	0	5	2	1	0	0	9	5
Omission errors	9	6	9	13	1	1	2	5
Total	9	11	11	14	1	1	11	10

### ChatGPT4 and Darwin Core field names

Out of 2128 herbarium labels fed to ChatGPT, it extracted 420 terms, of which 155 were valid Darwin Core terms and 265 were hallucinated. It should be noted that this was after a term cleanup pass was performed on the data and any changes to the case of the letters were ignored. Although ChatGPT hallucinated a large number of terms, the actual number of instances of fields across labels that used the hallucinated terms was not as large. There were 23,094 instances extracted, but only 1062 (4.6%) included hallucinated term labels. Hallucinations were unpredictable. Some were simple to correct, for example, one type of hallucination assumed that another namespace existed (e.g., “gbif:identificationRemarks”, although dwc:identificationRemarks is a valid term); in this case, the term name was converted to the correct one and used. Other hallucinations, however, were more esoteric, such as “QF”, for which it was necessary to examine the content associated with that term.

In addition to the issue of hallucinated terms, a key concern with ChatGPT4 is the formatting of the return. The returned JSON output was often improperly formatted with extra commas, improper quoting, the replacement or addition of extra characters, and the addition of extra text surrounding the JSON output. Of the 2128 labels that were processed, 503 (23.64%) had data formatting issues. The end result was that 503 (23.64%) had incorrectly formatted JSON output that could be salvaged, 542 (25.47%) labels had one or more hallucinated terms, and 112 (5.26%) labels contained both issues.

## DISCUSSION

This work showcases the power of using multiple approaches to produce digital data records that together help reduce errors that occur using any single method. In particular, we developed a RB‐NLP approach (LRBE) and tested how well it performed against a well‐used LLM, ChatGPT4. The LRBE approach has reasonably good precision and sometimes has excellent accuracy; critically, it will neither hallucinate names of fields or instance value data in those fields, and it always produces the exact same result on repeated uses. This approach is also easily adjusted when improvements are needed. However, the LRBE approach requires significant effort by an expert that understands both herbarium label construction and NLP tasks well enough to correct issues.

The need for significant effort to develop rule‐based information extraction is due to having to write one or often several rules for every field type and form. For instance, when extracting information about taxa, there are patterns for every commonly used taxon level and separate functions for when there is a binomial or trinomial term versus a monomial term. The taxon authority extraction builds on the bi‐, tri‐, or monomial term. This is then fed into a function that recognizes a binomial taxon followed by an authority that is then followed by a lower‐level term with its own authority (e.g., “*Neptunia gracilis* Muhl. ex Willd. var. *varia* (Nutt.) Brewer”). There are even other forms for taxon names, such as “*Neptunia gracilis* & *Mimosa sensitiva*”, or instances where one species is mentioned in relation to another (e.g., “It resembles *M. sensitiva* in amplitude”). Because of these factors, the development of a working LRBE is both time intensive and challenging. Furthermore, even with significant effort, error rates are still high. By contrast, ChatGPT4 requires a relatively simple set of prompt engineering approaches and can produce digital data records in Darwin Core format that contain fewer errors. However, ChatGPT4 is prone to other types of errors, e.g., non‐deterministic results and the potential for a profusion of terms that do not conform to existing standards.

Ensemble approaches using outputs from RB‐NLP and ChatGPT4 can help resolve both issues, as shown in our results. The ensemble approach leverages the strengths of LRBE in terms of mostly assembling the correct content into a predefined set of known Darwin Core fields. This provides a useful scaffold and framework to blend in the often‐superior extraction results from ChatGPT4. While it is possible that more careful prompt engineering could also improve ChatGPT4 results, it is challenging to understand a priori what prompts are required. Given that ChatGPT4 has multiple issues and that ensembling fixes a proportion of them simultaneously with little extra overhead, we advocate the value of this combined approach as a pragmatic step forward. Continuing advances in the quality of OCR of specimen labels, automated information extraction tools, and ensemble approaches will likely further reduce error rates, potentially to a point that rivals the quality achieved through human effort. However, based on the results here, more effort is needed to improve the quality of automated approaches; even in the best case of ensembling, half of the records scored still contained omission or commission errors. Even with continued improvements, we argue there will always be the need for expert validation. We have made the case elsewhere (Guralnick et al., 2024) of the importance of humans in the loop to improve both quality and model performance, and we advocate strongly for that approach here.

We note that our work here focuses mostly on core fields that are typically required for capture to enable best use of collections downstream for both research and collections management purposes. Herbarium labels often also report key traits of the specimen, such as flower color or leaf size. Here as well it can be challenging to capture traits successfully, even though LRBE has its origins in trait parsing. A next step will be testing how to best tune ensembling approaches for more performant assembly of the rich trait and interaction data on labels.

Assembling rich trait data cannot be fully decoupled from core field extraction. One of the overall challenges with label information extraction is that the more data a label contains, the easier it becomes to confuse an LRBE approach. The longer the label, the easier it is for extraction tools to pull the wrong content and associate it with the wrong term, although this is only one of many factors that likely impacts how well information extraction works. For instance, LRBE will sometimes mistake route numbers (e.g., “Rt. 12”) for a count. This can be counteracted by adding more rules that bar a count when it is preceded by a route abbreviation. However, trying to build rules for all possible vagaries of how labels are written is an impossible task, and the key goal is to find common errors of commission and reduce their rate as much as possible using such rules.

We close here with three key observations about the current state of automated label digitization and likely next steps. First, ChatGPT4 is a commercial solution and costs money to use. A longer‐term solution will be developing and deploying open‐source LLMs such as LLaMA (Touvron et al., 2023) that could replace ChatGPT4 or its successors. Such models can likely be tuned to perform better than a generalized LLM such as ChatGPT4. Second, our work has focused on typewritten labels that can be digitized using OCR. Future efforts could leverage rapidly advancing methods for handwritten text recognition (HTR) to improve digitization and information extraction for older, handwritten labels generated prior to the use of typewriters and computers. Finally, we believe the work here can be extended and utilized broadly for natural history collections digitization. One aspect of this extension is recognizing that automated approaches can be combined to deliver both digital data records and other insights, such as leaf traits, from specimens simultaneously (Ott and Lautenschlager, 2022; Weaver et al., 2023). More broadly, herbarium labels are some of the most verbose, commonly containing heterogeneous content, when compared across different types of natural history collections. By contrast, insect labels are typically far less wordy or heterogeneous, and therefore likely less error prone, for automated extraction approaches. Further efforts to test approaches across different types of labels and to build more production‐strength tools are critical next steps.

## AUTHOR CONTRIBUTIONS

All authors were involved in the conception of this research. R.L. developed the code and designed prompts for ChatGPT4, and R.P.G. and M.W.D. designed the validation approaches and developed statistical analyses. R.P.G. and J.M.A. acquired the funding. All authors were involved in writing initial drafts and editing the final version of the manuscript. All authors approved the final version of the manuscript.

## Supporting information


**Figure S1.** Plot of the predicted error rate based on label length for the LRBE results, based on the logistic regression model presented in the main text.
**Table S1.** Types of core field errors contained in the LRBE output.
**Table S2.** Types of core field errors contained in the ChatGPT output.

## Data Availability

All code is open source and available at https://github.com/rafelafrance/digi-leap. Data and scoring sheets used in creating and assessing models are available at https://zenodo.org/records/10642072.
