# Supplementary material for: Ensemble automated approaches for producing high‐quality herbarium digital records
Source: Appl Plant Sci. 2024 Nov 5;13(1):e11623. doi: 10.1002/aps3.11623 (PMC11788903; doi:10.1002/aps3.11623)
Supplement: Supplementary file 1 — Figure S1. Plot of the predicted error rate based on label length for the LRBE results, based on the logistic regression model presented in the main text. Table S1. Types of core field errors contained in the LRBE output. Table S2. Types of core field errors contained in the ChatGPT output. [file APS3-13-e11623-s001.docx]

**Supporting information for “Ensemble automated approaches for producing high-quality herbarium digital records”**

**Table S1.** Types of core field errors contained in the LRBE output. There were 193 errors in total, with significant errors in locality and recordedBy. Omissions in locality fields were often placed in recordedBy, leading to commission errors in that field. Not all core fields are present in each record. We use Darwin Core names for these field names.

|  | **Darwin Core field** | | | | | | | |
| --- | --- | --- | --- | --- | --- | --- | --- | --- |
| **Error type** | **recordedBy** | **record Number** | **eventDate** | **locality** | **scientificName** | **country** | **stateProvince** | **county** |
| Commission errors | 45 | 8 | 4 | 16 | 5 | 1 | 9 | 5 |
| Omission errors | 10 | 6 | 4 | 48 | 10 | 2 | 2 | 13 |
| Total | 55 | 14 | 8 | 64 | 15 | 3 | 11 | 18 |

**Table S2.** Types of core field errors contained in the ChatGPT output. There were 99 errors in total, with significant issues with omissions in recordNumber. We also note significant omissions in stateProvince and locality. Not all core fields are present in each record. We use Darwin Core names for these field names.

|  | **Darwin Core field** | | | | | | | |
| --- | --- | --- | --- | --- | --- | --- | --- | --- |
| **Error type** | **recordedBy** | **record Number** | **eventDate** | **locality** | **scientificName** | **country** | **stateProvince** | **county** |
| Commission errors | 2 | 1 | 1 | 0 | 0 | 2 | 1 | 0 |
| Omission errors | 9 | 35 | 2 | 17 | 0 | 2 | 14 | 13 |
| Total | 11 | 36 | 3 | 17 | 0 | 4 | 15 | 13 |


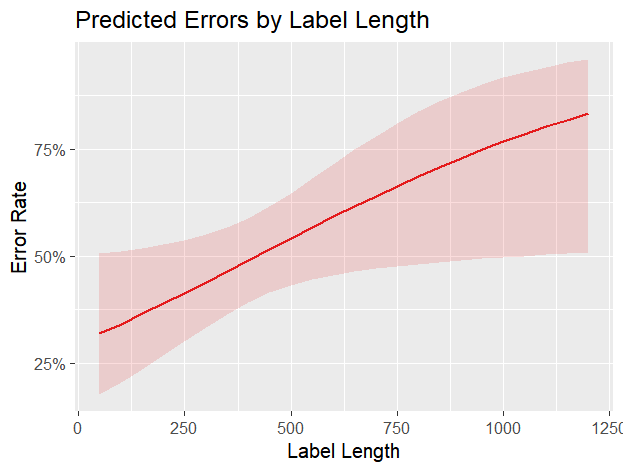


**Figure S1.** Plot of the predicted error rate based on label length for the LRBE results, based on the logistic regression model presented in the main text.
